# Fasciclin-Like Arabinogalactan-Protein 16 (FLA16) Is Required for Stem Development in *Arabidopsis*

**DOI:** 10.3389/fpls.2020.615392

**Published:** 2020-12-11

**Authors:** Edgar Liu, Colleen P. MacMillan, Thomas Shafee, Yingxuan Ma, Julian Ratcliffe, Allison van de Meene, Antony Bacic, John Humphries, Kim L. Johnson

**Affiliations:** ^1^School of BioSciences, University of Melbourne, Parkville, VIC, Australia; ^2^CSIRO, Agriculture and Food, CSIRO Black Mountain Science and Innovation Park, Canberra, ACT, Australia; ^3^La Trobe Institute for Agriculture and Food, Department of Animal, Plant and Soil Sciences, La Trobe University, Bundoora, VIC, Australia; ^4^Sino-Australia Plant Cell Wall Research Centre, College of Forestry and Biotechnology, Zhejiang A & F University, Hangzhou, China

**Keywords:** cell wall, glycoprotein, fasciclin-like arabinogalactan-protein, stem, biomechanics

## Abstract

The predominant Fascilin 1 (FAS1)-containing proteins in plants belong to the Fasciclin-Like Arabinogalactan-protein (FLA) family of extracellular glycoproteins. In addition to FAS1 domains, these multi-domain FLA proteins contain glycomotif regions predicted to direct addition of large arabinogalactan (AG) glycans and many contain signal sequences for addition of a glycosylphosphatidylinositol (GPI)-anchor to tether them to the plasma membrane. FLAs are proposed to play both structural and signaling functions by forming a range of interactions in the plant extracellular matrix, similar to FAS1-containing proteins in animals. FLA group B members contain two FAS1 domains and are not predicted to be GPI-anchored. None of the group B members have been functionally characterized or their sub-cellular location resolved, limiting understanding of their function. We investigated the group B *FLA16* in *Arabidopsis* that is predominantly expressed in inflorescence tissues. *FLA16* is the most highly expressed *FLA* in the stem after Group A members *FLA11* and *FLA12* that are stem specific. A FLA16-YFP fusion protein driven by the endogenous putative *FLA16* promoter in wild type background showed expression in cells with secondary cell walls, and FLA16 displayed characteristics of cell wall glycoproteins with moderate glycosylation. Investigation of a *fla16* mutant showed loss of FLA16 leads to reduced stem length and altered biomechanical properties, likely as a result of reduced levels of cellulose. Immuno-labeling indicated support for FLA16 location to the plasma-membrane and (apoplastic) cell wall of interfascicular stem fiber cells. Together these results indicate FLA16, a two-FAS1 domain FLAs, plays a role in plant secondary cell wall synthesis and function.

## Introduction

Arabinogalactan-proteins (AGPs) are cell wall glycoproteins with highly complex and diverse structures with potential roles in signaling. Some of the many roles attributed to AGPs include hormone signaling, cell expansion and division, embryogenesis of somatic cells, differentiation of xylem, responses to abiotic stress, plant growth and development (Seifert and Roberts, 2007; Ma et al., 2018). These studies suggest that they are multifunctional, similar to what is found in mammalian proteoglycans/glycoproteins (Filmus et al., 2008; Schaefer and Schaefer, 2010; Tan L. et al., 2012). The Fasciclin-Like AGP (FLA) sub-class of AGPs are characterized by the presence of Fasciclin1 (FAS1) domains in addition to the AGP motifs in their protein backbones. FAS1 proteins were initially identified in grasshopper growth cones and fasciculating axons (Bastiani et al., 1987). Since this discovery FAS1 domains have been identified in proteins across all kingdoms from a broad spectrum of taxonomic classifications, including insects (Elkins et al., 1990), humans (Horiuchi et al., 1999; Gonzalez-Gonzalez and Alonso, 2018), bacteria (Ulstrup et al., 1995; Moody and Williamson, 2013), fungi (Miyazaki et al., 2007), algae (Huber and Sumper, 1994), and plants (Johnson et al., 2003; Seifert, 2018; Shafee et al., 2020). Fasciclin domains are associated with cell adhesion functions and occur predominantly in proteins predicted to be located in the extracellular space. FAS1-containing proteins play critical roles in the structure, function and development of organisms (Seifert, 2018). In humans, periostin and transforming growth factor-β-induced protein (TGFβI) are two of the best characterized FAS1 domain-containing proteins due to their medical implications in conditions such as wound responses, bone regeneration, corneal dystrophies, and cancer (Mosher et al., 2015; Walker et al., 2016; Gonzalez-Gonzalez and Alonso, 2018).

In plants, FLAs are the predominant FAS1-containing proteins and may play parallel roles to that in animals with both structural and signaling functions (Johnson et al., 2003). The presence of either single and/or multiple AGP or FAS1 domains in FLAs opens the potential for a range of putative interactions, similar to that which occurs in mammalian FAS1 proteins. Support for some FLAs being glycosylated with AG glycans was shown by their interaction with β-glucosyl Yariv reagent that selectively precipitates AGPs (Johnson et al., 2003; Sato et al., 2018). Immunoprecipitation of *Arabidopsis* FLA4 showed it can be labeled with AGP-specific antibodies and is observed at a higher molecular weight than predicted by the protein backbone alone (Xue et al., 2017). The AG-glycans are likely to display structural heterogeneity, thereby providing enormous potential to act in molecular recognition and signaling pathways (Baldwin et al., 2001; Tan H. et al., 2012). FLAs are also predicted to be post-translationally modified to include *N*-glycosylation in the FAS1 domain and many FLA members predict a C-terminal GlycosylphosphatidylInositol (GPI)-anchor. GPI-anchors lead to attachment to the outer leaflet of the plasma membrane facing the apoplastic space/cell wall (Muniz and Zurzolo, 2014), have the potential to be cleaved by phospholipases to release the proteins into the extracellular space (Udenfriend and Kodukula, 1995; Borner et al., 2003), and are implicated in signaling pathways. Support for FLAs in the apoplast is shown by proteomic data of adherent and non-adherent seed mucilage that identified FLA10 and FLA17 (Tsai et al., 2017) and FLA4-citrine was detected in the mucilage pocket prior to mucilage extrusion (Griffiths et al., 2016). Sub-cellular localization of FLA4-CFP in roots and GFP-FLA3 in hypocotyl cells of *Arabidopsis* suggest they are primarily located at the plasma membrane and that the GPI-anchor influences this location (Li et al., 2010; Xue et al., 2017), suggesting release into the apoplast can be both tissue and developmental dependent. By means of pair-wise sequence comparisons of the predicted proteins, the 21 *Arabidopsis* FLAs were grouped into 4 sub-groups (A-D) based on structural similarity (Johnson et al., 2003). All group A and C members are predicted to be GPI-anchored whereas all of group B and three members of group D are not predicted to be GPI-anchored. Detailed bioinformatic characterization of FLA sequences has revealed further distinguishing features within these groups, including 19 fasciclin and 10 distinct AG types (Shafee et al., 2020). A strong correlation between sequence features was found, for example, the fasciclin domain type strongly predicts *N*-glycosylation site position and GPI anchor presence and is also highly predictive (45%) of the type of AG glycomotifs (Shafee et al., 2020). The group B FLAs in *Arabidopsis* (FLAs 15, 16, 17, 18) were found to contain fasciclin types R-H which has a strong co-occurrence with specific types of AG and non-AG regions. None of the group B members have been functionally characterized and the role of these glycoproteins in the apoplast remains unknown. Additionally, the specific functions and potential cooperation between the different domains in FLAs remains largely unexplored (Shi et al., 2003; MacMillan et al., 2010; Johnson et al., 2011). A study of FLA4 has provided the first comprehensive study of the functional domains (Xue et al., 2017). Removal of the GPI-anchor did not affect function, the C-terminal (but not N-terminal) FAS1 motif was essential for function and the *O*- and *N*-glycosylation impacted molecular trafficking (Xue et al., 2017).

Bioinformatic studies have identified FLA sequences throughout the plant kingdom, from algae to eudicots. FLAs belong to multigene families, for example, 21 FLA members have been identified in the *Arabidopsis* genome, 24 in rice (*Oryza sativa*), 34 in wheat (*Triticum aestivum*), and 18 in *Eucalyptus* (*Eucalyptus grandis*) (Schultz et al., 2002; Johnson et al., 2003; Faik et al., 2007; MacMillan et al., 2015; Ma et al., 2017). The function of FLAs has largely been inferred through phenotypes attributed to the respective *fla* mutants. In *Arabidopsis*, *fla1* mutants have reduced ability to undergo shoot regeneration in *in vitro* experiments (Johnson et al., 2011), RNA interference and overexpression of *FLA3* suggest a specific role in pollen development (Li et al., 2010), a salt overly sensitive 5 (*sos5*) mutant was identified as a mutation in the gene encoding FLA4 (Shi et al., 2003), and *fla9* mutants show increased seed abortion in response to drought (Cagnola et al., 2018). Glycosylation of FLA4 has been proposed to be important for mediating root growth (Xue et al., 2017). In plants, one proposed function of the AG glycans of AGPs is to cross-link wall polysaccharides (Kjellbom et al., 1997). This was confirmed for ARABINOXYLAN PECTIN ARABINOGALACTAN PROTEIN1 (APAP1) which was shown to be covalently cross-linked to both pectins and arabinoxylans (Tan et al., 2013). When and how frequently such attachments occur is unclear and requires much more knowledge of both the glycan and fasciclin domain structures of individual AGPs. FLAs are suggested to influence the organization of cell wall polysaccharides such as cellulose and pectins leading to alterations in wall properties and impacts on plant growth (MacMillan et al., 2010; Griffiths et al., 2016). FLAs are also suggested to interact with signaling proteins such as RLKs to activate signaling pathways to ensure wall integrity (Basu et al., 2016). Genetic interaction studies of *fla4/sos5* and mutants in the cell wall receptor-like kinases, FEI1/FEI2 suggest they function in a linear pathway during root development (Basu et al., 2016; Griffiths et al., 2016).

A subset of FLAs have been shown to be specifically expressed in cells with secondary walls in stems and fibers in several different species. The secondary walls of plant stems provide mechanical strength that enable plants to maintain an upright habit. Factors that influence stem strength are of interest for reducing crop losses in windy and stormy conditions and/or for enhancing fiber quality in textile and wood industries. *Arabidopsis FLA11* and *FLA12* (group A members) were shown to be the most highly expressed of all FLAs in the inflorescence stem (MacMillan et al., 2010) and homologs identified in other species such as *Eucalyptus*, *Zinnia* and *Populus* also show predominant expression in the stem (Dahiya et al., 2006; Dharmawardhana et al., 2010; MacMillan et al., 2015). Studies of the stem-specific FLAs suggest they function in maintaining stem biomechanical properties via regulation of wall architecture and/or biosynthesis (MacMillan et al., 2010, 2015; Wang et al., 2015). FLAs have also been shown to regulate non-lignified secondary walls, with functions in elongation and strengthening in cotton seed fibers and the bast fibers in hemp stems (Huang et al., 2013; Guerriero et al., 2017; MacMillan et al., 2017).

Investigation of *fla11fla12* double mutants showed a reduction in tensile strength and stiffness of stems, increased cellulose microfibril angle (MFA) and reduced cellulose, galactose and arabinose content (MacMillan et al., 2010). Despite differences in the biomechanical properties in stems, no overall change in plant morphology was observed in *fla11fla12* mutants suggesting further redundancy with other FLA members. Amongst all the FLAs, *FLA16*, is considered a good candidate given it is the most highly expressed *FLA* in the stem after *FLA11* and *FLA12* (MacMillan et al., 2010). Since the biological role(s) of FLA16 in *Arabidopsis* are unknown, in this study, a *fla16* mutant was investigated to determine if FLA16 plays a role in stem development.

## Materials and Methods

### Sequence Analysis

Fasciclin-like arabinogalactan-protein sequences within the R-H family (as defined in Shafee et al., 2020) were isolated from the sequence set described in that work. The Fasciclin domains (regions matching the Pfam PF02469 Hidden Markov Model) of those sequences were extracted. A separate phylogeny was generated for the R-type and for the H-type domains [JTT + I + G4 model identified via IQtree ModelFinder (Kalyaanamoorthy et al., 2017); Maximum-likelihood, 1000-bootstrap tree calculated via RaxML (Stamatakis, 2014)]. The two phylogenies were compared via a tanglegram generated by Dendroscope3 (Huson and Scornavacca, 2012).

### Plant Material and Growth Conditions

Wild type *Arabidopsis thaliana* (Columbia-0 ecotype; WT) and *fla16* (SALK_131248) were obtained from the Arabidopsis Biological Resource Centre (ABRC). Plants were grown in either soil in jiffy pots (Garden City, Australia) or on 1/2 MS plates in long day conditions, 16 h light/8 h dark at 21°C in controlled growth chambers (Conviron, Canada) with 50% humidity and fluorescent lighting 190 ± 30 μmol m^–2^ s^–1^. Analysis of stem morphology (see Table 1) was determined in 68 biological replicates (total stem and first internode length) or 33 biological replicates (transverse stem area) for *fla16* and WT. Stem length measurements of *fla16* complementation lines (*pFLA16*:FLA16 and *pFLA16*:FLA16-VH) was determined for four independent transformed lines with 10 biological replicates for each line.

**TABLE 1:** Measurement of stem morphology of WT and *fla16* stems at maturity^*a*^.

	WT	*fla16*	*n*
**Stem length (cm)**			
Total stem	46.44 (±0.66)	**35.02^*b*^ (±0.38)**	68
First internode	11.79 (±0.34)	**5.46 (±0.26)**	68
First internode/total stem (%)	25.55 (±0.73)	**15.73 (±0.78)**	68
**Stem transverse area (mm^2^)**			
Basal stem	0.87 (±0.04)	**0.75 (±0.03)**	33
First node stem	0.97 (±0.05)	**0.67 (±0.04)**	33
First node/basal stem (%)	119.55 (±7.09)	**97.00 (±4.39)**	33

*^a^Growth stage 6.5 as outlined in Boyes et al. (2001). ^b^Bold text in numeral data column indicates data value statistically significant at p < 0.05 using a Student’s t test. n presents number of biological replicates. Bracket indicates ± SE.*

### Dark Grown Hypocotyl and Isoxaben Treatment

*Arabidopsis* seeds were plated on 1/2 MS plates either containing the herbicide isoxaben (Sigma #36138) (2 nM) or plates lacking isoxaben (control). The plates were wrapped three times in aluminum foil sheets to block light and stratified at 4°C for 3 days prior to removal of the foil and light treatment for 4 h at room temperature (RT). The plates were then re-covered in foil and transferred to a growth chamber and maintained in the dark at 21°C for 4 days. Three replicate plates were investigated for each treatment with 40–60 individual plants investigated for each line.

### Quantitative Real Time PCR (Q-PCR)

RNA was isolated from approximately 100 mg of fresh plant material using a RNeasy Plant Mini Kit (Qiagen 74904). Investigation of *FLA16* transcripts in WT tissues were taken from plants at growth stage 6.5. Q-PCR analysis of *FLA16* and *CESAs* in stem tissue was investigated in the basal 10 cm stem of plants at growth stage 6.5. DNase treatment was carried out using a DNase I kit (Invitrogen #18068-015) and complementary DNA (cDNA) synthesized from 1 μg of RNA using a SuperScript III Reverse Transcriptase kit (Invitrogen #18080-093). An absolute quantitative method adapted from Burton et al. (2004) was used with three biological and three technical replicates. For quantification of *FLA16* transcripts in *pFLA16*:FLA16 and *pFLA16*:FLA16-VH complementation and reporter lines, two independent transformed lines with three biological repeats were investigated. Primers are listed in Supplementary Table S4. Transcript levels were normalized with three housekeeping genes, *Tubulin*, *Cyclophylin*, and *Glyceraldehyde 3-phosphate dehydrogenase* (*GAPDH*). DNA standards for each primer were prepared according to Burton et al. (2004). Q-PCR reactions were performed with the KAPA SYBR FAST Q-PCR Kit Master Mix (2×) Universal (Kapa Biosystems, #KK4601) in a Bio-Rad CFX384 Real-Time System (Bio-Rad).

### Generation of YFP Protein Fusion Constructs

Fasciclin-like arabinogalactan-protein 16 complementation and reporter constructs were generated using Seamless Cloning with a NEBuilder^®^HiFi DNA Assembly kit (New England BioLabs #E5520) as per the manufacturer’s instructions. Primers used for DNA amplification are listed in Supplementary Table S4 and cloned into the pGreen II vector. The putative *FLA16* promoter included a 2341bp fragment upstream of the *FLA16* coding region. Overlapping with the *FLA16* coding region and the promoter region closest to the start codon, is a natural antisense transcript (AT2G35859). Constructs were introduced into *Arabidopsis* using the floral-dip method as outlined in Weigel and Glazebrook (2006). Transformed plants were selected using BASTA. Between 3 and 5 independent transformed lines were verified using BASTA selection and genotyping until homozygous for the transgene (T3 generation). For lines with YFP fusion constructs, protein blotting and imaging of YFP fluorescence were also used to validate transformants.

### Confocal Laser-Scanning Microscopy

Fluorescence microscopy of fresh tissue, either whole mount or transverse sections, was carried out on a Leica SP5 microscope (Leica Microsystems, Germany). Laser beam lines exciting at 514 nm were employed for the VENUS yellow fluorescent protein (YFP), and fluorescence emitted between 520 and 530 nm was captured. Images were analyzed with Leica LAS Lite software and images processed with Fiji (Schindelin et al., 2012). Three independent *pFLA16*:FLA16-VH transformed lines and three biological repeats for each line were imaged.

### Protein Extraction and Blotting

Stem tissue (approximately 5 g from 50 plants for two independent transformed *pFLA16*:FLA16-VH lines) was homogenized in protein extraction buffer (100 mM Tris–HCl pH 7.4, 150 mM NaCl, 1% NP-40 and cOmplete Protease inhibitor, Roche # 11697498001) in an ice bath, with a ratio of tissue to buffer of 0.5 g/mL. The homogenate was rotated end-to-end at 4°C for 1 h. The mixture was filtered through Miracloth (Merck Millipore #475855) and the liquid centrifuged at 10,000 *g*, at 4°C for 10 min to pellet cell wall extracts. The supernatant was used for mixed membrane extraction. Cell wall material was resuspended in protein extraction buffer (Wall), centrifuged again and resuspended in protein extraction buffer with 1% SDS (Wall SDS).

Mixed membranes (MMs) were prepared according to Doblin et al. (2009), the supernatant after centrifugation (100,000 *g*) constituted the cytoplasmic fraction. Microsomes were re-suspended in a minimal amount of 50 mM Tris–HCl (∼0.5–1 mL) containing complete EDTA-free proteinase inhibitor (Roche #11836170001). Microsomes were then resuspended in additional buffer containing 1% SDS. FLA16-HV proteins were enriched using the immunoaffinity of YFP to bind anti-GFP antibodies coupled to agarose beads (GFP-Trap, Chromotek, #gta-20). Proteins were separated on Mini-PROTEIN^®^ TGX Stain-Free^TM^ Gels (Bio-Rad #456-8094) and subsequently transferred in an iBlot^®^ 2 Gel Transfer Device (Thermo Fisher #IB21001) to a nitrocellulose membrane (Thermo Fisher #IB23001). FLA16-VH detection was performed with anti-GFP primary antibody (Chromotek #GTA-10) and goat anti-mouse horse radish peroxidase (HRP) conjugated secondary antibody (Thermo Fisher #31430). Chemiluminescent signals were detected by a ChemiDoc^TM^ MP Imaging System (Bio-Rad #170-8280).

### Histology

*Arabidopsis* stem tissues were hand-sectioned to a thickness of approximately 2 mm and fixed according to the protocol outlined in Wilson et al. (2012). Stems embedded in LR white were sectioned (500 μm) and stained with toluidine blue as previously described (Chateigner-Boutin et al., 2014). Hand-sections of fresh stems (approx. 0.5 mm) were stained with Toluidine blue (0.5%) solution and Mäule staining (Pradhan Mitra and Loque, 2014).

Fixed stem sections were examined under a Leica DM6000 B compound microscope (Leica Microsystems, Germany) and images captured by a Leica DFC450 C camera (Leica Microsystems, Germany). Images were analyzed using Image J (Schneider et al., 2012). For area measurements the stem circumference was traced, and area calculated using Image J plugin. For tissue area measurements transverse sections of fixed material were divided into several regions including: (1) cortex, (2) pith, and (3) intra-vascular region which consisted of interfascicular fiber, phloem, cambium, and xylem cells. These were traced and area calculated using Image J for six biological replicates with two technical replicates each line. Cell number was determined by counting the total number of cells in transverse sections of 4–6 independent biological replicates with two technical replicates each. All cells (1000–1200) within a stem transverse section (imaged at x200) were counted for the different tissue types. Cell size was determined by dividing the tissue area by the number of cells. Cell wall thickness of IF fibers was determined from transverse sections of fixed material by measuring the length between adjacent IF cells from lumen to lumen and dividing by two. Eighty wall lengths were measured for three biological samples with two technical replicates each.

### Transmission Electron Microscopy

The protocol for preparation of plant cells for TEM was adapted from Wilson and Bacic (2012). Stems embedded in LR White were sectioned using a Leica UC7 Ultramicrotome (Leica Microsystems, Germany) to a thickness of 90 nm. Sections were collected on formvar coated copper grids (Microscopy Solutions, Australia). Grids were stained using a mouse anti-6x-His tag primary antibody (Thermo Fisher #14-6657-80) diluted 1:100 (control grids with no primary antibody were also prepared), and 18 nm goat anti mouse 18 nm gold conjugated secondary antibody (Jackson ImmunoResearch #115-215-146) at 1:10 dilution. Grids were post-stained using 2% uranyl acetate for 5 min and Reynold’s lead citrate for 1 min.

The grids were viewed using a Joel JEM-2100 transmission electron microscope equipped with a Gatan Orius SC 200 CCD camera. Image analysis was performed with ImageJ software to measure gold density. The amount of gold per 1 μm^2^ was measured as the total number of gold in the cytoplasm or cell wall divided by the area. For PM, 1 μm^2^ was calculated as the area 0.05 μm either side of the PM. Two independent *pFLA16*:FLA16-VH transformed lines with two biological replicates each were investigated.

### Chemical Analysis of Alcohol-Insoluble Residue (AIR) Cell Wall Material

Alcohol-insoluble residue material was prepared as described in Pettolino et al. (2012) which was adapted from Sims et al. (1996) as well as Kim and Carpita (1992). The acetic/nitric protocol for analysis of crystalline cellulose was adapted from Updegraff (1969). Linkage analysis was performed as outlined in Pettolino et al. (2012). Two (*fla16*) or three (WT) biological replicates were analyzed with two technical replicates each for linkage and acetic/nitric determination. Crystalline cellulose determination in stems of complementation lines of the *fla16* mutant with *pFLA16*:FLA16 or *pFLA16*:FLA16-VH were analyzed in two independent transformed lines with three biological replicates each.

### Measurement of Biomechanical Properties of the Stem

Three-point flexural and tensile and tests were carried out by a 4500 series Instron universal testing machine (series IX automated materials testing system)^1^ as described by MacMillan et al. (2010). The modulus of elasticity (stiffness) was calculated using Hooke’s law whereas flexural three-point bending stiffness and strength were calculated via standard equations (MacMillan et al., 2010). Tensile strength was calculated as the maximum load needed to break the stem within the gauge length, and this was divided by the cross-sectional area of the stem (MacMillan et al., 2010). Six or twelve biological repeats were investigated for each line.

## Results

### *FLA16* Is Predominantly Expressed in Inflorescence Stems

**FIGURE 1:**
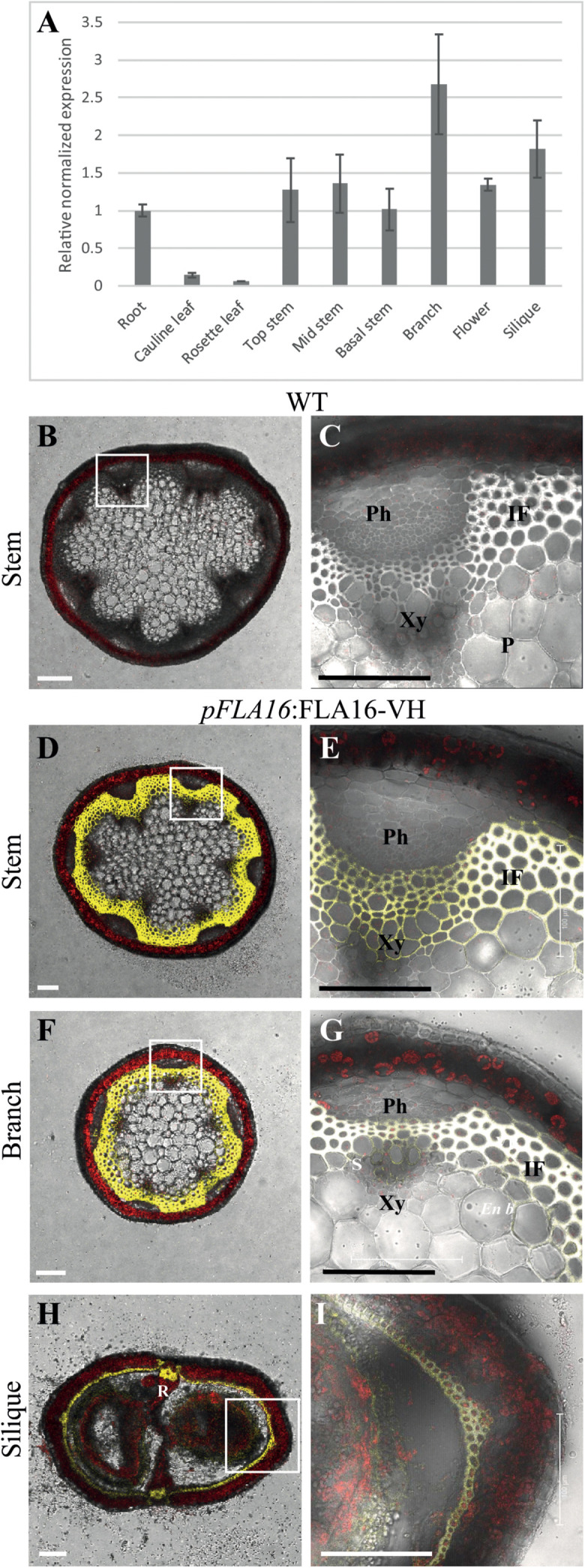
Transcript levels of *FLA16* and visualization of *pFLA16:FLA16-VH* in stems of *Arabidopsis*. **(A)** Q-PCR analysis of *FLA16* transcript levels in *Arabidopsis* tissues at maturity (growth stage 6.5) (Boyes et al., 2001). Transcript levels were determined relative to DNA standards of known concentration and normalized with GAPDH, tubulin, and cyclophilin housekeeping genes (Czechowski et al., 2005). *N* = 2 independent experiments with two biological replicates, data represented as mean ± SE. **(B–G)** Visualization of YFP in WT **(B,C)** and plants expressing *pFLA16:FLA16-VENUS-HIS (VH)* in WT background. Representative images are shown based on investigation of three independent transformed lines. Merged image of YFP, autofluorescence (red) and light channels in transverse sections of mature stems at the first internode **(B–E)**, branch **(F,G)**, and fully elongated silique **(H,I)**. White boxes indicate position of tissue magnified in **(C,E,G,I)**. YFP signal was observed in cells with secondary cell walls, including interfascicular fiber (IF) and xylem (Xy) in stems and branches. White signal in **(E,G)** shows saturated signal. In siliques YFP is observed in the endocarp *b* (*En b*) layer, vascular bundle (VB) and replum (R) regions, as well as the seed coat (S). Scale bar = 100 μm.

Group B members contain fasciclin domain types R-H, with R being one of the most basal fasciclin domain types and present in algal FLAs. Fasciclin type H first appeared in land plants (embryophytes) and is almost exclusively associated with type R (Shafee et al., 2020). In addition to the very strong co-evolution of R and H fasciclin domains, robust association of specific types of AG and non-AG regions also occurs in the Group B FLAs and this is very stable compared to other FLA members (Shafee et al., 2020). Phylogenetic analysis of fasciclin types R-H confirmed the stability of the relationship in a diverse range of plant species (Supplementary Figure S1). Group B FLAs in *Arabidopsis* consists of four members, FLA15, FLA16, FLA17, and FLA18. The expression profiles of *FLA15, FLA16*, and *FLA18* (*FLA17* not available) in the *Arabidopsis* Atlas eFP browser (Klepikova et al., 2016) shows they are expressed throughout plant development. *FLA16* was the most highly expressed group B *FLA* in stem tissue (Supplementary Figure S2) and given FLAs have previously been shown to function in stem biomechanics (MacMillan et al., 2010, 2015) we chose to investigate FLA16 in more detail. *FLA16* was expressed in a range of tissues at low levels with highest expression in the hypocotyls of young seedlings, stem, flowers and silique. To confirm the levels of *FLA16* transcript abundance, Q-PCR was undertaken using a range of *Arabidopsis* tissues. These included the root, rosette leaf, cauline leaf, basal-, mid- and upper-region of stems, flower and silique (Figure 1A). Transcripts of *FLA16* were predominantly found in inflorescence tissues, including the stem, branch, flower and silique.

To facilitate visualization and biochemical analyses of FLA16, YFP fusion proteins were generated. The predicted protein backbones of FLA16 consists of a domain structure with a signal peptide, 2 FAS domains, a central AGP-like domain and no GPI signal sequence (Supplementary Figure S3). The coding region of *FLA16* was fused to the enhanced yellow fluorescence protein variant, Venus (V) and poly-histidine (HIS/H) sequences at the C-terminus. This construct was driven by the predicted endogenous *FLA16* promoter (*pFLA16:*FLA16-VH) (Supplementary Figure S3).

*Arabidopsis* wild-type (WT) plants expressing *pFLA16:*FLA16-VH were examined using confocal microscopy to visualize YFP fluorescence. In transverse-sections of fresh stems from WT, no YFP signal was detected (Figures 1B,C). In *pFLA16:*FLA16-VH lines, a strong YFP signal was seen in cells with secondary walls such as the interfascicular fibers, xylem vessels, sclereids within the phloem, and endodermal cells adjacent to interfascicular fibers (Figures 1D,E). No YFP signal was detected in cells with primary walls, that is, the epidermis, cortex and pith (Figures 1D,E). The expression of *pFLA16:*FLA16-VH was also investigated in other tissues. In branches, a strong YFP signal was observed in cells with secondary walls, similar to that seen in the main stem (Figures 1F,G). Transverse sections of mature siliques showed YFP present in the endocarp *b* layer and replum (Figures 1H,I). A weak YFP signal was also seen in *pFLA16*:FLA16-VH lines in the developing seeds (Figure 1I). These results suggest FLA16 is predominantly located in cells with secondary walls in inflorescence tissues.

### Identification and Characterization of a *fla16* Mutant

A T-DNA insertion in the intron of the *FLA16* gene was identified in the *Arabidopsis* SALK collection (SALK_131248) (Supplementary Figure S4A). The insertion in *FLA16* was confirmed using PCR genotyping and Q-PCR of *FLA16* transcripts in the stem tissue. The expression levels of *FLA16* in the *fla16* mutant was 21% of the wild-type (WT) levels suggesting this was a severe knock-down mutant line (Supplementary Figure S4B). The *fla16* mutant was investigated for phenotypic differences in growth and development compared to WT plants. The timing of selected growth stages was analyzed in *fla16* compared to WT plants according to the method of Boyes et al. (2001). No obvious differences in growth of seedlings was observed (Supplementary Figure S5A). The number of rosette leaves and timing of bolting and appearance of the first flower were assessed. At flowering, *fla16* had fewer rosette leaves and an earlier bolting time of 3 days but showed no difference in the time of first flower emergence (Supplementary Figure S5B and Supplementary Table S1).

**FIGURE 2:**
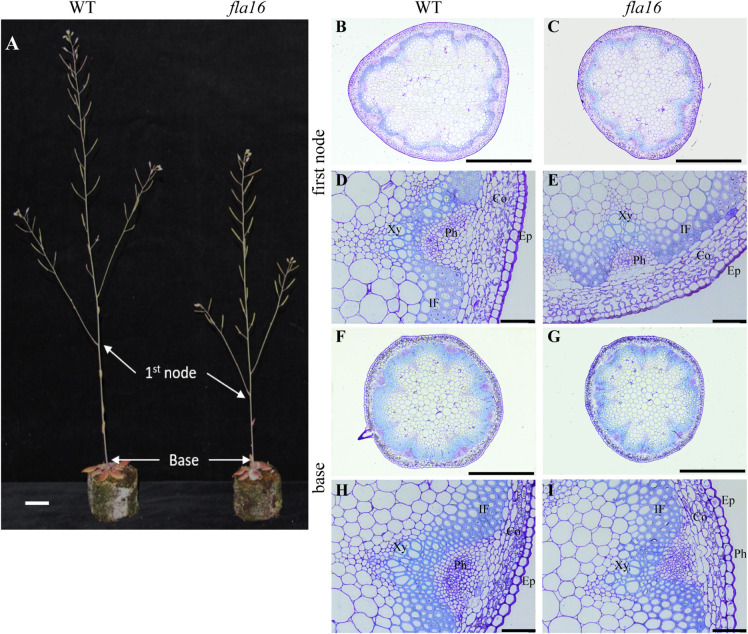
Stem phenotypes of WT and *fla16* mutants at maturity. **(A)** Representative image of WT and *fla16* mutants grown to post-maturity [growth stage 6.9 (Boyes et al., 2001)] shows the reduced total stem and first internode lengths of *fla16* compared to WT. Toluidine blue staining of transverse sections of stems of WT **(B,D,F,H)** and *fla16* **(C,E,G,I)** at the base and first internode in mature stems (growth stage 6.5) shows the reduced stem diameter in *fla16* compared to WT and no obvious differences in cellular organization. Epidermis (Ep), cortex (Co), phloem (Ph), interfascicular fiber (IF), and Xylem (Xy). Scale bar = 2 cm in **(A)**, 500 μm in **(A,B,F,G)** and 50 μm in **(D,E,H,I)**.

Due to the expression of *pFLA16:*FLA16-VH in fiber cells in stems/branches, the stem length and area of *fla16* mutants was of interest. Length of the total stem and first internode were measured in plants at maturity (growth stage 6.5) grown in long day conditions (Table 1). For area measurements, the base and first node positions were chosen as two developmentally comparable regions. The *fla16* mutant was shown to develop a shorter stem with a significant reduction in the length of both the total stem and first internode (Table 1 and Figure 2). The average total stem length of *fla16* mutants was reduced by 25%, and the first internode length was reduced by 54% compared to WT. In addition to changes in the stem length, *fla16* mutants have thinner stems. The average stem area of *fla16* mutants was reduced by 14% and 31% at the base and first node, respectively, compared to WT (Table 1).

Transverse sections of fresh tissue from the base and first node of the stem were stained with toluidine blue and Mäule’s staining for cell wall polysaccharides and lignin, respectively, to determine if any obvious morphological differences in either primary or secondary cell wall morphology occurs in *fla16* compared to WT (Supplementary Figure S6). In addition, histological analyses of transverse sections of fixed stem material (growth stage 6.5) were undertaken (Figure 2A). No obvious cellular morphological differences between *fla16* and WT were observed. Analyses of tissue area in transverse sections revealed that pith area was significantly reduced in the base (30%) and first node (38%) of *fla16* compared to WT, whereas no significant difference was detected in cortex area. The *fla16* stem showed a significantly reduced vascular region area [consisting of interfascicular fibers (IF), phloem, cambium, and xylem cells; 32%] at the first node but not the base (Table 2).

**TABLE 2:** Analysis of tissue and cellular morphology in mature stems of WT and *fla16* plants^*a*^.

	WT	*fla16*	*n*
**Tissue area (mm^2^)**			
***Base***			
Cortex	0.18 (±0.01)	0.17 (±0.01)	6
Vascular region^*b*^	0.33 (±0.01)	0.32 (±0.02)	6
Pith	0.37 (±0.03)	**0.26 (±0.02)**	6
***First node***			
Cortex	0.169 (±0.01)	0.15 (±0.01)	4–6
Vascular region	0.31 (±0.02)	**0.21 (±0.01)**	4–6
Pith	0.5 (±0.05)	**0.31 (±0.04)**	4–6
**Cell size (μm^2^)**			
***Base***			
Epidermal	238.94 (±20.71)	226.41 (±19.54)	5–6
IF	128.76 (±1.62)	130.65 (±7.84)	5–6
Cortex	150.98 (±18.77)	204.02 (±10.74)	5–6
Xylem vessel	184.70 (±7.49)	210.12 (±10.53)	5–6
Pith	817.14 (±36.15)	742.14 (±23.42)	5–6
***First node***			
Epidermal	151.71 (±10.41)	154.97 (±5.00)	4–5
IF	142.30 (±6.24)	140.30 (±6.77)	4–5
Cortex	138.68 (±20.07)	124.02 (±4.27)	4–5
Xylem vessel	171.98 (±5.79)	198.79 (±7.91)	4–5
Pith	1140.12 (±50.49)	**902.80 (±47.11)**	4–5
**Cell number**			
***Base***			
Pith	492.33 (±38.19)	**360.40 (±22.80)**	5–6
IF	1149.75 (±39.86)	1130.25 (±87.10)	4
Xylem vessel	922.75 (±49.57)	884.75 (±54.15)	4
***First node***			
Pith	462.33 (±10.30)	**365.80 (±13.31)**	4–5
IF	811.75 (±47.57)	**635.00 (±50.86)**	4
Xylem vessel	646.75 (±59.09)	618.25 (±25.39)	4
**Wall thickness (μm)**			
IF Base	0.057 (±0.001)	0.053 (±0.001)	5–6
IF First node	0.067 (±0.002)	0.069 (±0.002)	5–6

*^a^Growth stage 6.5 as outlined in Boyes et al. (2001). ^b^Vascular region includes IF and vascular bundles. Values represented as mean (±SE), bold text indicates data value statistically significant at p < 0.05 using Student’s t test. n represents number of biological replicates, each with two technical replicates. All cells (300–1200) for each tissue type were counted for each replicate.*

Analysis of cell size for epidermal, cortex, IF, xylem vessel, and pith cells were undertaken using transverse sections. Significantly smaller pith cells in the first node but not the base was observed for *fla16* mutants. Other cell types examined, including epidermal, xylem vessel, and IF cells, did not show statistically significant differences in size at both stem positions. A count of the average cell number from five transverse sections was determined for the pith, interfascicular fiber and xylem vessel cells (Table 2). The total number of pith cells observed in the base and first node revealed *fla16* had significantly fewer pith cells than WT. In addition, the *fla16* stem showed a significant reduction in the number of IF cells at the first node. This suggested that the reduction in pith number in *fla16* compared to WT is the major cause of the reduced stem area at the base (Table 2). At the first node, reduced cell size and number of pith cells as well as reduced number of interfascicular fiber cells contributes to a reduced stem area. As FLA16 is expressed in cells making secondary walls, contains a N-terminal secretion signal and is not predicted to be GPI-anchored, the expected location is in the apoplast. The *fla16* mutant was therefore investigated for differences in IF wall thickness. The thickness of walls between IF cells closest to the cortex was calculated for the base and first node. No differences in wall thickness was observed (Table 2).

### FLA16 Is Moderately Glycosylated and Present in Secondary Cell Walls in the *Arabidopsis* Stem

**FIGURE 3:**
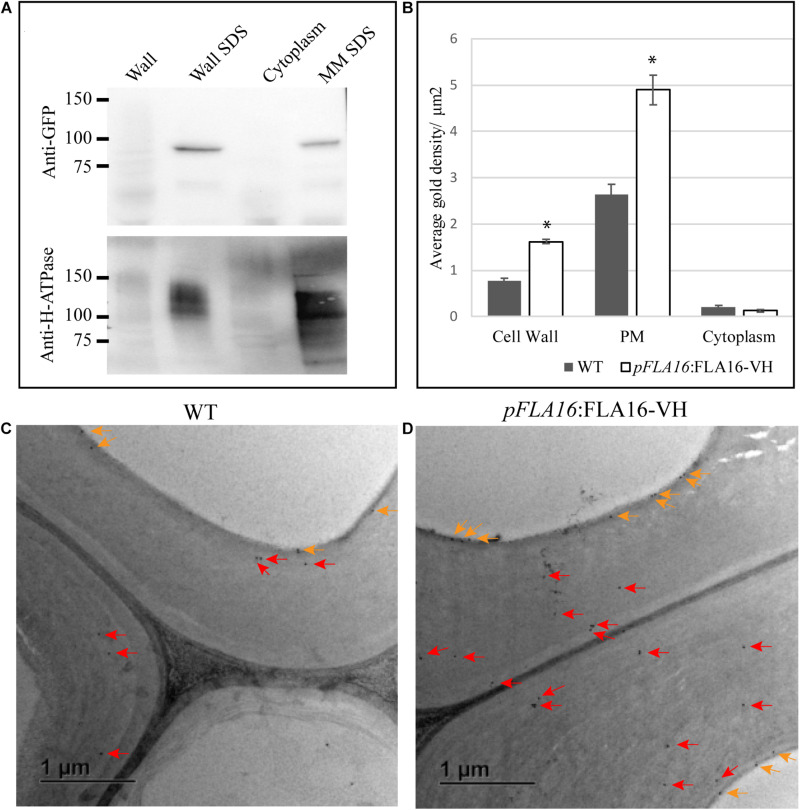
Detection of FLA16-VH fusion proteins in *Arabidopsis* stems. **(A)** Proteins extracted from stems were fractionated into wall, SDS wall extract (Wall SDS), microsomal membranes extracted with SDS (MM SDS) and cytoplasm. FLA16-HV is detected in both Wall SDS and MM SDS fractions using an anti-GFP antibody **(upper panel)**. The Wall SDS and MM SDS fractions are enriched for Plasma Membrane (PM) as shown by replicate western blots probed with the anti-H-ATPase antibody bound in both. **(B)** Average gold density per 1 μm^2^ of interfascicular fiber cell wall, plasma membrane (PM) or cytoplasmic region for anti-HIS antibody in ultrathin sections of the stem at the first internode. Significantly higher amounts of gold labeling (*p* < 0.05 using Student’s *t* test, indicated by *) are observed in the wall and PM of pFLA16:FLA16-HV plants compared to WT with no difference in the cytoplasm. *n* = 5–8 cells from two biological replicates. Error bars indicate standard error, *p* < 0.05 using *t*-test. **(C,D)** Transmission electron micrograph images of interfascicular fiber cells at the stem first internode of WT **(C)** and *pFLA16*:FLA16-VH **(D)**. Gold labeling in the cell wall (red arrow) and plasma membrane (orange arrows) are indicated.

The post-translational modifications (PTMs) and sub-cellular localization of FLA16 was investigated in WT plants expressing *pFLA16:*FLA16-VH. As FLA16 is not predicted to contain a GPI-anchor, but does contain a predicted signal peptide, the expected location of FLA16 would be in the cell wall. Proteins extracted from stem tissue were separated into wall, microsomal/mixed membrane (MM), and cytoplasmic fractions. An antibody raised against the PM localized H-ATPase (Hayashi et al., 2010) was used to investigate PM enrichment and an anti-GFP antibody that cross reacts with YFP (see “Materials and Methods”) was used to detect the presence of the FLA16-VH fusion proteins. FLA16-VH was detected in wall and MM fractions when SDS was present in the extraction buffers (Figure 3A). SDS was used to solubilize proteins that may be bound in the wall or membrane (Feiz et al., 2006). FLA16-VH was not detectable in the cytoplasmic fraction. The anti-H-ATPase antibody showed the H-ATPase PM marker was present in SDS fractions from both the wall and MM. This is due to cross-contamination of PM in wall fractions (Cho et al., 2015; de Michele et al., 2016; Durufle et al., 2017; Figure 3A). The size of FLA16-VH proteins detected on Western blots was approximately 90 kDa with an additional faint band at 60 kDa (Figure 3A). The predicted size of FLA16-VH proteins is 74.7 kDa. This suggests the 90 kDa form contains PTMs and the 60 kDa is potentially a proteolytically processed form of the FLA16-VH. The protein encoded by *FLA16* predicts two *N-*linked glycans, one in each of the fasciclin domains, and 13 sites for attachment of *O*-linked glycans, 10 of these occurring between the two fasciclin domains (Supplementary Figure S3). The increased size of FLA16 is expected to result from addition of *N*-glycans and *O*-linked AGs.

To further investigate the sub-cellular location of FLA16-VH, immunogold labeling using an anti-HIS antibody was undertaken of stem sections taken from the first internode and visualized using transmission electron microscopy (TEM). Consistent with protein extraction and blotting analysis, FLA16-VH appears to be largely associated with cell wall and PM with significantly more gold labeling observed in the walls and PM of interfascicular fiber cells in *pFLA16*:FLA16-VH plants than WT (Figures 3B–D). Background levels of HIS labeling was expected based on native extracellular HIS-rich proteins, including HIS-rich AGPs (Liu and Mehdy, 2007; Callaway and Singh-Cundy, 2019). Low levels of gold labeling was observed in the cytoplasm of *pFLA16*:FLA16-VH fiber cells, with similar amounts seen in the WT. Little to no gold labeling was observed in sections incubated with no primary antibody controls (Supplementary Figure S7). These data indicate FLA16 is moderately glycosylated and located at the PM and wall of cells with secondary cell walls.

### Carbohydrate Content and Biomechanics Are Altered in *fla16* Stems

**FIGURE 4:**
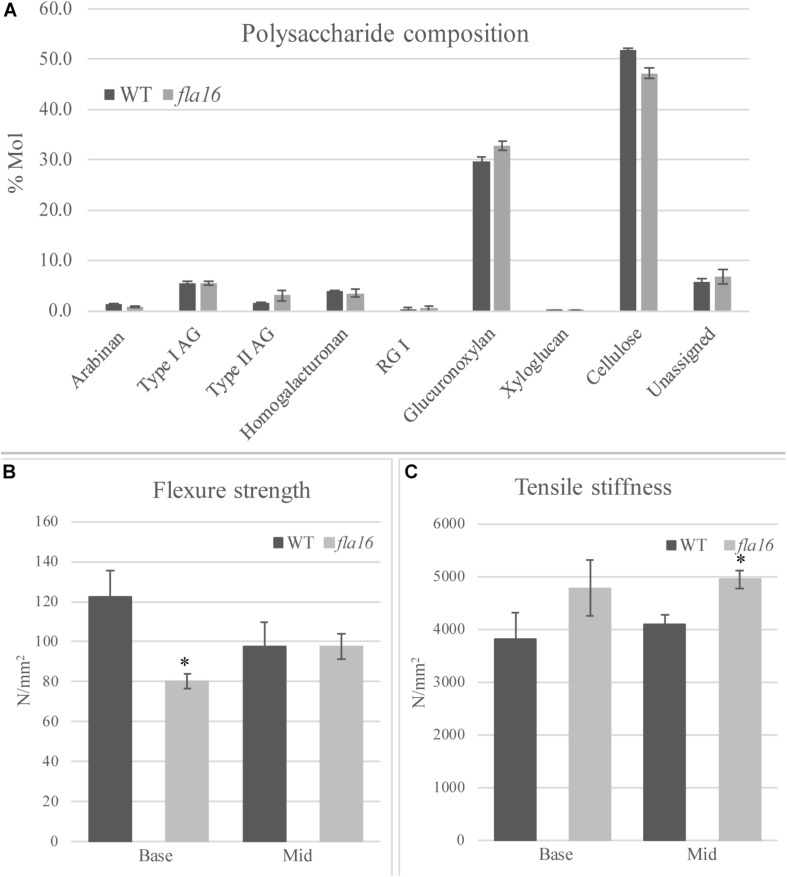
Cell wall polysaccharide composition derived from linkage analysis and biomechanical tests of mature stems of *fla16* and WT. **(A)** Polysaccharide linkage analysis shows a reduction of cellulose and an increase in glucuronoxylan in *fla16* compared to WT with no difference in the other polysaccharides. *N* = 2–3 biological replicates. For full data see Supplementary Table S3. Biomechanical tests on the base- and mid- stem show significantly decreased flexure strength **(B)** in the base stem of *fla16* compared to WT and increases in tensile stiffness **(C)**. *N* = 6–12 stems, *p* < 0.05 (^∗^) using a Student’s *t* test. Data are presented as means ± SE.

In order to determine whether cell wall compositional changes occur in the *fla16* mutant stems, polysaccharide composition was examined by linkage analyses of the alcohol insoluble residue (AIR) representing the cell wall fractions extracted from *fla16* and WT stems (growth stage 6.9) (Moller et al., 2012). Linkage analyses of the cell wall polysaccharides revealed an approximately 9% reduction of cellulose and 10% increase in glucuronoxylan in *fla16* stems (Figure 4A and Supplementary Table S2). The reduction in cellulose was also investigated using an acetic/nitric cellulose assay to determine the amount of crystalline cellulose (Updegraff, 1969) which recorded an approximately 16% reduction in stems of *fla16* mutants (Supplementary Table S3). As changes in cell wall composition can influence the biomechanical properties of the stem, this was investigated using biomechanical tests.

Mature, dry stems were divided into 12 cm segments and biomechanical tests were conducted on the base- and mid- stem. The upper region was excluded due to the reduced stem length of *fla16* mutants. Dried stems were used to avoid differences in hydration that can lead to variable results (Molina-Freaner et al., 1998). Flexure strength and tensile stiffness tests were undertaken. The strength indicates the maximum load required to break the stem whereas stiffness is a measure of the elasticity of the stem (MacMillan et al., 2010). Flexure tests revealed that the basal stems of *fla16* have significantly reduced strength compared to WT (Figure 4B). No significant differences in flexure strength of the mid-stems was found between *fla16* and WT (Figure 4B and Supplementary Table S3). Tensile strength tests of base- and mid-stems identified a significantly increased stiffness in the mid-stems, with a similar trend in the base-stems although this is not significantly different to WT (Figure 4C and Supplementary Table S3).

### The *fla16* Mutant Is More Sensitive to Isoxaben Treatment Than WT and Has Altered *CESA* Transcript Levels

**FIGURE 5:**
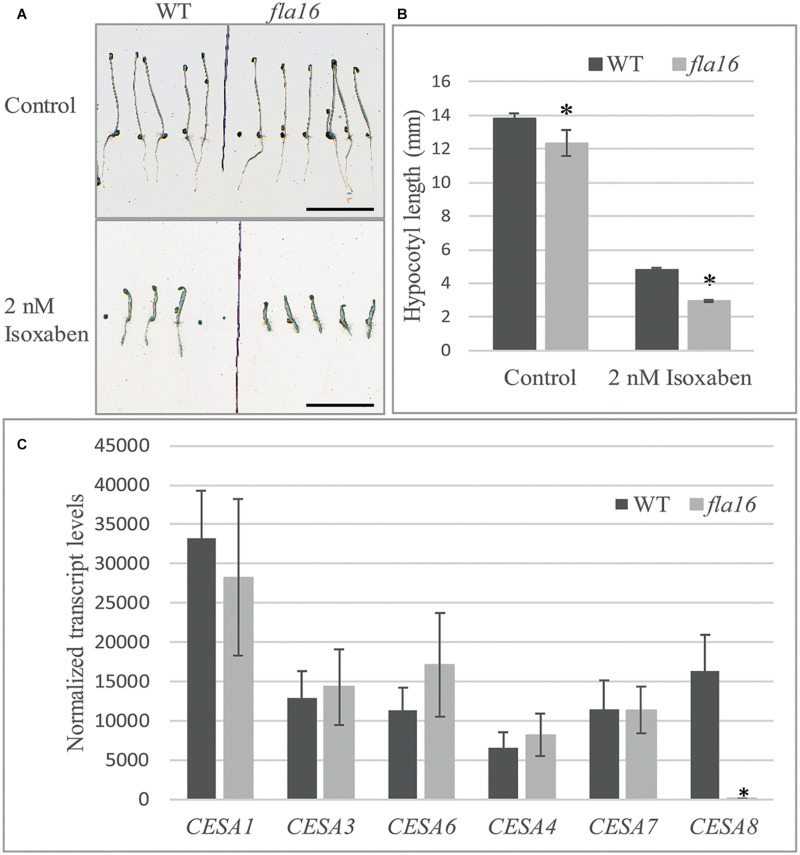
Isoxaben treatment of dark grown *Arabidopsis fla16* and WT seedlings and Q-PCR analysis of CESA transcript levels. Significantly reduced hypocotyl length is observed in *fla16* seedlings on control plates and those treated with 2 nM isoxaben compared to WT **(A,B)**. *N* = 40–60, data represented as mean ± SE, * indicates statistically significant value, *P* < 0.05 using one-way ANOVA. **(C)** Q-PCR analyses of transcript levels of *CESAs* involved in primary (*1*, *3*, and *6*) and secondary (*4*, *7*, and *8*) wall biosynthesis shows *CESA8* levels are significantly reduced in the basal stem segments of *fla16* mutants compared to WT. Transcript levels were determined relative to DNA standards of known concentration and normalized with *GAPDH* and *tubulin* (Czechowski et al., 2005). *N* = 3 biological replicates, data represented as mean ± SE, * indicates statistically significant value *p* < 0.05 using one-way ANOVA.

Compared to WT, mutants with reduced cellulose levels frequently display higher sensitivity to the herbicide isoxaben, as evidenced by reduced hypocotyl growth and swelling (Desprez et al., 2002). Isoxaben has been shown to inhibit cellulose synthesis in plant primary walls (Desprez et al., 2002). *FLA16* is expressed in dark grown hypocotyls (Winter et al., 2007) therefore WT and *fla16* mutants were tested for sensitivity to isoxaben during dark-grown seedling development. Hypocotyl growth of *fla16* mutants was found to be significantly reduced compared to WT, in both control (89% of WT hypocotyl length) and isoxaben (60% of WT hypocotyl length) treatments (Figure 5B). The *fla16* mutant was more sensitive to isoxaben treatment than WT with a 4.4 fold reduction in hypocotyl length compared to a three fold reduction in WT, relative to the control (Figure 5A).

Stem tissue was investigated to determine if transcript levels of *CESA1*, *CESA3* and *CESA6*, involved in synthesis of cellulose in primary walls, and *CESA4*, *CESA7*, and *CESA8* in secondary walls was altered in *fla16* compared to WT using Q-PCR analysis. No significant differences in transcript levels of *CESA1*, *CESA3*, and *CESA6* were observed between *fla16* and WT base stems (Figure 5C). *CESA8* showed a dramatic reduction in transcript levels in *fla16* stems compared to WT in the base-stem, whereas *CESA4* and *CESA7* levels were unchanged (Figure 5C).

### Complementation of the *fla16* Mutant

Two *FLA16* complementation constructs driven by the endogenous *FLA16* promoter were generated and transformed into the *fla16* mutant; *pFLA16:*FLA16 included only the *FLA16* coding region and *pFLA16:*FLA16-VH as previously described (Supplementary Figure S3). Homozygous plant lines for the transgenes in the *fla16* mutant background were generated and examined for *FLA16* expression by Q-PCR and complementation of reduced cellulose content and stem length.

The expression of *FLA16* transcripts in the total stem of *fla16* was 21% of that observed in WT (Supplementary Figure S4). In the *fla16 pFLA1*6*:*FLA16 and *fla16 pFLA16:*FLA16-VH lines the transcript levels were 73% and 123% of WT levels, respectively. Based on acetic/nitric cellulose assays, the cellulose content in *fla16 pFLA16:*FLA16 and *fla16 pFLA16:*FLA16-VH stems was 98% and 93%, respectively, of WT levels (Supplementary Figure S8). This is a significant increase in crystalline cellulose compared to the *fla16* line whose content was reduced to 83% of WT. Both complementation lines partially recovered the stem length phenotype compared to WT whereas the length of first internode was not recovered and was comparable to *fla16* (Supplementary Figure S8).

## Discussion

Fasciclin-like arabinogalactan-proteins belong to large, multigene families in plant species and are regulated at both the transcriptional and post-translational levels. Studies of stem-specific FLAs containing a single FAS1 domain (Group A, fasciclin type O) in *Arabidopsis*, *Eucalyptus*, *Zinnia*, and *Populus* suggest they function in maintaining stem biomechanical properties via regulation of wall architecture and/or biosynthesis (Dahiya et al., 2006; MacMillan et al., 2010, 2015; Wang et al., 2015, 2017). In this study we reveal a novel role for FLA16, belonging to the group B FLAs with fasciclin type R-H, in stem biomechanics and regulation of cellulose levels. Expression of *FLA16* is predominantly in cells with secondary walls, including in stems, which overlaps with that of *FLA11* and *FLA12*, previously shown to regulate stem biomechanics in *Arabidopsis* (MacMillan et al., 2010). Transcriptional studies of secondary wall development in *Eucalyptus grandis* (MacMillan et al., 2015), *Populus trichocarpa* (Wang et al., 2015), cotton fiber formation in *Gossypium hirsutum* (MacMillan et al., 2017), and tension wood formation in *Populus tremula* (Bygdell et al., 2017) show group B FLA members have highest expression in these tissues. This suggests that a co-ordinated balance of different FLAs is required to maintain cell wall integrity. It remains to be determined if group B FLA members are involved in both primary and secondary wall development. During stem development, growth must be carefully co-ordinated with maintaining structural integrity. Therefore, secondary wall deposition is balanced with stem growth and likely involves feedback mechanisms to ensure tissue integrity. FLA16 could potentially play a role to delay flowering or promote vegetative growth as fewer rosette leaves and an earlier bolting time was observed in *fla16* lines. Here we provide evidence that FLA16 is localized in the cell wall and plasma membrane-wall interface of cells with secondary walls (Figure 3). A reduced stem diameter and pith cell number was found in *fla16* stems compared to WT. A reduction in cellulose and altered biomechanics in *fla16* suggests wall integrity is compromised, most likely in fiber and xylem tissues where YFP was observed in *pFLA16*:FLA16-VH lines (Figure 1). Partial recovery of the *fla16* mutant phenotypes was observed using two complementation constructs. A natural antisense transcript overlaps the *FLA16* promoter and coding region and all group B members in *Arabidopsis* have a conserved intron at the 3′ end of the sequence. Greater understanding of the regulation of *FLA16* at the transcriptional and post-transcriptional level is required to determine how this might influence function.

Cellulose is the major component of secondary walls and a key factor influencing mechanical properties (Turner and Somerville, 1997). In this study, reduced cellulose levels are observed in *fla16* mutants and particularly in crystalline cellulose. The crystallinity of cellulose can be modified by a number of factors, including proteins that associate with the cellulose synthase complex (CSC) and cellulose-matrix phase interactions. For example, the GPI-anchored COBRA-like proteins can directly bind cellulose and regulate microfibril crystallinity in *Arabidopsis* (Roudier et al., 2002), rice (Liu et al., 2013), and maize (Sindhu et al., 2007), and mutants have reduced mechanical strength. The expression of *FLA16* in xylem and fiber cells of the stem and its location in the wall suggests that the reduced stem length and altered biomechanics observed in *fla16* are related to altered architecture and/or composition of secondary walls in stems. The reduced flexural strength in *fla16* basal stems is consistent with reduced cellulose content and increased tensile stiffness in the *fla16* middle stem could also result from changes in composition of the wall matrix (i.e., lignin and hemicelluloses) (Kohler and Spatz, 2002) and wall architecture, in particular the cellulose microfibril angle (MFA) (Cave and Walker, 1994; Keckes et al., 2003). A small increase in the hemicellulose glucuronoxylan (GX) in *fla16* may also contribute to the altered stem properties as GX makes physical contacts with cellulose in secondary walls (Bromley et al., 2013; Grantham et al., 2017). A role for FLA16 in regulation of primary walls during early development cannot be ruled out given secondary wall cellulose synthesis is not affected by isoxaben (Watanabe et al., 2018) and *fla16* dark grown seedlings displayed increased sensitivity to isoxaben treatments. In inflorescence tissues FLA16 appears to be restricted to tissues with secondary walls and transcript profiling showed primary wall *CESAs* were unchanged in *fla16* mutants. Genes strongly co-expressed with *FLA16* include *CESA5* and *FLA15* (Obayashi et al., 2018). CESA5 makes a major contribution to cellulose production in seed mucilage (Sullivan et al., 2011). It is possible that FLA16 interacts with different complexes/signaling pathways depending on the tissue and cellular context. In stem tissue only *CESA8* showed reduced transcript levels in *fla16*. Mutations in *CESA8* were identified as *irregular xylem* (*irx1*) mutants due to a collapsed xylem phenotype (Taylor et al., 2000). A reduction in stem cellulose content of up to 40% is seen in *irx1* mutants compared to WT and altered biomechanical properties (Turner and Somerville, 1997; Taylor et al., 2000). The reduction in *CESA8* transcripts could contribute to the *fla16* mutant phenotype. As *fla16* does not display a collapsed xylem phenotype and only a moderate reduction in cellulose, it suggests some CESA8 is still functional. It is unclear how reduced levels of *FLA16* influence transcription of *CESA8*. FLAs have the potential to interact via protein-protein, protein-carbohydrate and carbohydrate-carbohydrate associations and could therefore be multifunctional, acting in both signaling pathways and wall structure. Roles in cell wall integrity sensing pathways that detect changes in wall mechanics to regulate growth or stress responses have been proposed (Wolf et al., 2012). This is supported by genetic studies suggesting FLA4/SOS5 interacts with the leucine-rich repeat-RLKs FEI1 and FEI2 to maintain seed mucilage adherence (Xu et al., 2008; Griffiths et al., 2016; Engelsdorf et al., 2018).

Roles in extracellular sensing and signaling would be consistent with the role of FAS1-containing proteins in animals. For example, Periostin in humans interacts with structural proteins such as fibronectin, tenascin and collagen, as well as activating intercellular signaling through Nephroblastoma Overexpressed (NOV/CCN3) and NOTCH1 pathways (Tanabe et al., 2010; Kii and Ito, 2017; Takayama et al., 2017). In addition to the FAS1 domains, AG-glycans in FLAs could potentially bind wall polysaccharides such as hemicelluloses and pectins as has been shown for the AGP, APAP1 (Tan et al., 2013). The exact mechanism of how FLA16 influences either biosynthesis or deposition of cell wall components remains to be elucidated. The association of AGPs with cellulose in flax fiber secondary walls led to the proposal that AGPs might be secreted to the cell surface along with CESAs and then associate with cellulose in the thickening wall (Girault et al., 2000; Seifert and Roberts, 2007). Identification of interacting proteins and/or polysaccharides of FLA16 is needed to determine its role in secondary wall development and stem biomechanics.

To date, analysis of the functional domains of FLAs is restricted to FLA4/SOS5 and showed the N-terminal FAS1 domain to be critical in assisting PM localization, independently from the GPI-anchor, and the C-terminal FAS1 is required for function in roots (Xue et al., 2017). Biochemical and TEM results suggest FLA16 is located in the secondary cell wall and at the plasma membrane. FLA16 is not predicted to contain a GPI-anchor and so could associate with PM located integral membrane proteins or an alternative form of lipid attachment, such as S-acylation. The domain architecture of the group B FLAs to which FLA16 belongs is one of the most stable throughout plant evolution. Group B FLAs have fasciclin types R and H, which are almost exclusively associated with each other and specific types of AG regions and represented the largest group of FLAs in 18 plant proteomes (Shafee et al., 2020). The strong association of these domains suggests the function of these FLAs is relatively constrained.

## Conclusion

This research has provided new insights into the understanding of role of FLA16 in stem development. Our study supports FLA16 being in the secondary wall and containing AG-glycans. Functional studies testing the ability of FLA16 homologs from embryophyte ancestors to complement the *fla16* mutant, as well as mutant studies of further R-H type FLAs in *Arabidopsis* and other species, would provide knowledge of the functional conservation of this group and enhance understanding of the role of these glycoproteins. Domain deletion/swapping studies within FLA16/group B FLAs would be helpful to interpret the functional domains of the molecule. Data from the analyses of the *fla16* mutant has revealed a novel role for FLA16 in stem biomechanics and regulation of cellulose levels. This research provides a platform for further investigations toward uncovering functional mechanism(s) of these fascinating molecules during secondary wall development.

## Data Availability Statement

The datasets presented in this study can be found in online repositories. The names of the repository/repositories and accession number(s) can be found in the article/Supplementary Material.

## Author Contributions

EL performed the experiments, processed, analyzed, and interpreted the data, and wrote the manuscript with contributions from the other authors. JH, KJ, and AB assisted with supervision of EL and assisted with the interpretation of the data and the writing of the manuscript. EL and YM performed the protein extraction and blotting studies, prepared plant material, and assisted in data analysis. TS undertook sequence and phylogenetic analysis of group B FLAs. CM performed the biomechanical tests, processed and interpreted the data, provided feedback on the manuscript, and collaborated with KJ in conceiving the original research question. AM and JR performed the TEM studies and provided feedback on the manuscript. KJ conceived the original research plan, supervised EL and assisted with the design and interpretation of the data, and wrote the article with EL and JH. All authors contributed to the article and approved the submitted version.

## Conflict of Interest

The authors declare that the research was conducted in the absence of any commercial or financial relationships that could be construed as a potential conflict of interest.

## Footnotes

1 http://www.instron.co.uk

## Supplementary Material

Supplementary Figure 1 Phylogeny of fasciclin domain types R-H in Group B FLAs.

Supplementary Figure 2 Expression level of *FLA15*, *FLA16* and *FLA18* in selected *Arabidopsis* tissues.

Supplementary Figure 3 Protein sequence and predicted domains of *Arabidopsis* FLA16 and schematic of FLA16-YFP fusion constructs.

Supplementary Figure 4 Schematic of the T-DNA insertion in *FLA16* and transcript levels of *FLA16* in stems of *Arabidopsis*.

Supplementary Figure 5 Phenotyping of WT and *fla16* mutants at selected growth stages.

Supplementary Figure 6 Cellular morphology in transverse sections of WT and *fla16* mature stems stained with toluidine blue and *Mäule* reagent.

Supplementary Figure 7 Transmission electron micrograph image of interfascicular fiber cells at the stem first internode of *pFLA16*:FLA16-VH.

Supplementary Figure 8 Complementation of the *fla16* mutant with *pFLA16:FLA16* and *pFLA16:FLA16-VH*.

Supplementary Table 1 Analysis of selected growth stages of long day grown wild-type (WT) and *fla16* plants.

Supplementary Table 2 Polysaccharide composition from stems of *fla16* and WT deduced from linkage analysis.

Supplementary Table 3 Measurement of stem biomechanical properties and cellulose content of WT and *fla16* stems at maturity.

Supplementary Table 4 Primers for Q-PCR and cloning.

Data File 1 Sequences, alignments and phylogenies included in analysis.
