# Supplementary material for: Fasciclin-Like Arabinogalactan-Protein 16 (FLA16) Is Required for Stem Development in Arabidopsis
Source: Front Plant Sci. 2020 Dec 11;11:615392. doi: 10.3389/fpls.2020.615392 (PMC7758453; doi:10.3389/fpls.2020.615392)
Supplement: Supplementary Figure 1 — Phylogeny of fasciclin domain types R-H in Group B FLAs. [file Data_Sheet_2.PDF]

Supplementary Material

Liu et al., Fasciclin-like Arabinogalactan-protein 16 (FLA16) is required for stem development in *Arabidopsis*

1. Supplementary Tables

Supp Table S1. Analysis of selected growth stages[a] of long day grown wild-type (WT) and *fla16* plants.

	WT	*fla16*	*N*
**Rosette number at flowering**	13.78 (± 0.17)[c]	**12.06 (± 0.32)**	5
**Bolting (day)**	26.16 (± 0.55)	**23.73 (± 0.34)**	48-50
**First flower emergence (day)**	30.48 (± 0.51)	30.08 (± 0.34)	48-50

[a]Growth stages as outlined in Boyes et al. (2001b).
Bold text indicates a statistically significant value at $p<0.05$ using a student's t test. Bracket indicates ± SE. N = biological replicates

Supp Table S2. Polysaccharide composition (mol%) from stems of *fla16* and WT deduced from linkage analysis[a]

		WT				*fla16*		
		**1**	**2**	**3**	**Mean (± SE)**	**1**	**2**	**Mean (± SE)**
**Arabinan**	1,5-Ara (f)	1.1	1.8	1.0		0.8	0.7	
	**Total Arabinan**	**1.1**	**1.8**	**1.0**	**1.3 (± 0.3)**	**0.8**	**0.7**	**0.8 (± 0.1)**
**Type I AG**	1,4-Gal (p)	3.2	2.5	3.4		4.0	3.1	
	1,4,6-Gal (p)	1.3	1.2	1.5		0.9	1.2	
	t-Ara (f)	1.1	1.2	1.1		0.9	0.8	
	**Total Type I AG**	**5.6**	**4.9**	**6.0**	**5.5 (± 0.3)**	**5.9**	**5.0**	**5.5 (± 0.5)**
**Type II AG**	1,6-Gal (p)	0.1	0.0	0.0		0.0	0.1	
	1,3,6-Gal (p)	0.7	0.5	1.7		2.0	1.2	
	1,3,4,6-Gal (p)	0.0	0.0	0.0		0.0	0.1	
	t-Ara(f)	0.0	0.5	0.0		2.0	0.0	
	t-Gal (p)	0.6	0.0	0.2		0.0	0.6	
	t-GlcA (p)	0.1	0.0	0.0		0.1	0.0	
	**Total Type II AG**	**1.5**	**1.0**	**1.9**	**1.5 (± 0.3)**	**4.1**	**2.0**	**3.1 (± 1.1)**
**HG**	1,4-GalA (p)	4.4	3.6	3.4		2.7	4.2	
	**Total HG**	**4.4**	**3.6**	**3.4**	**3.8 (± 0.3)**	**2.7**	**4.2**	**3.5 (± 0.7)**
**RG I/II**	1,4-GalA (p)	0.4	0.2	0.0		0.0	0.4	
	1,2,4-Rha (p)	0.4	0.2	0.0		0.0	0.4	
	**Total RG I/II**	**0.8**	**0.3**	**0.0**	**0.4 (± 0.2)**	**0.0**	**0.8**	**0.4 (± 0.4)**
**Glucuronoxylan**	1,4-Xyl (p)	25.9	25.2	25.3		27.0	27.9	
	1,2,4-Xyl (p)	2.5	2.5	1.5		2.4	2.9	
	t-GlcA (p)	2.5	2.5	1.5		2.4	2.9	
	**Total Glucuronoxylan**	**30.8**	**30.3**	**28.3**	**29.8 (± 0.8)**	**31.9**	**33.7**	**32.8 (± 0.9)**

Supp Table S2 continued.

**Xyloglucan**	1,4,6-Glc (p)	tr[b]	tr	tr		tr	tr	
	1,4-Glc (p)	tr	tr	tr		Tr	tr	
	1,2-Xyl (p)	tr	tr	tr		Tr	tr	
	t-Xyl (p)	tr	tr	tr		Tr	tr	
	**Total Xyloglucan**	**0.1**	**0.0**	**0.0**	**0.00 (± 0.00)**	**0.0**	**0.1**	**0.1 (± 0.1)**
**Cellulose**	1,4-Glc (p)	51.4	51.8	52.5		46.3	48.2	
	**Total Cellulose**	**51.4**	**51.8**	**52.5**	**51.9 (± 0.3)**	**46.3**	**48.2**	**47.3 (± 1.0)**
	**Total**	**95.7**	**93.8**	**93.1**	**94.2 (± 0.78)**	**91.7**	**94.7**	**93.2 (± 1.5)**
**Unassigned linkage**	t-Ara (f)	0.0	0.0	0.0		0.4	0.0	
	t-Xyl (p)	1.0	2.0	0.9		4.2	1.0	
	1,2-Xyl (p)	1.3	2.0	2.0		1.4	2.2	
	t-Glc (p)	0.1	0.0	0.0		0.0	0.2	
	1,3,4-Glc (p)	0.3	0.7	1.1		0.4	0.6	
	1,2,4-Glc (p)	0.4	0.4	0.5		0.0	0.4	
	1,3,4,6-Glc (p)	0.1	0.3	0.4		0.0	0.0	
	1,2,4-Man (p)	0.1	0.0	0.0		0.9	0.1	
	t-Gal (p)	0.0	0.3	0.0		0.0	0.0	
	t-GlcA (p)	0.9	0.5	1.8		0.9	0.8	
	**Total**	**4.2**	**6.2**	**6.7**	**5.7 (± 0.8)**	**8.2**	**5.3**	**6.75 (± 1.45)**

[a] data used to generate Figure 4A
[b] value is lower than 0.05 mol%.
For each plant line (biological replicate) the average of two technical replicates is shown
AG = Arabinogalactan, RG = Rhamnogalacturonan, HG = Homogalacturonan

Supp Table S3. Measurement of stem biomechanical properties and cellulose content of WT and *fla16* stems at maturity[a]

	WT	*fla16*	*N*
**Stem biomechanics (N/mm^2)**[b]			
**Flexure**			
Basal stem flexure strength	122.26 (± 13.23)	**80.32 (± 3.52)**	6
Basal stem flexure stiffness	7789.83 (± 755.89)	7992.82 (± 470.94)	6
Mid stem flexure strength	97.59 (± 12.38)	97.44 (± 6.38)	12
Mid stem flexure stiffness	14544.73 (± 1777.28)	13462.14 (± 926.87)	12
**Tensile**			
Basal stem tensile strength	83.13 (± 10.86)	92.54 (± 13.90)	6
Basal stem tensile stiffness	3822.34 (± 484.33)	4799.72 (± 529.22)	6
Mid stem tensile strength	74.55 (± 6.19)	68.43 (± 5.37)	12
Mid stem tensile stiffness	4098.26 (± 172.54)	**4952.03 (± 170.86)**	12
**Cellulose content (%)**[c]	50.54 (± 1.38)	**42.30 (± 0.18)**	3

[a] Growth stage 6.9 as outlined in Boyes et al. (2001a).
[b] N/mm^2 represents Newton/square millimetre.
[c] Crystalline cellulose content determined by the acetic/nitric digestion assay at growth stage 6.5 (Updegraff 1969).
Values represented as mean (± SE); bold text indicates statistically significant value at $p<0.05$ using student's t test. *N* represents number of biological replicates.

Supp Table S4. Primers for Q-PCR and cloning

Gene	Accession	Primer name	Sequence
**Q-PCR**			
Cyclophilin	AT2G36130	Cyclophilin qF	TGGCGAACGCTGGTCCTAATACA
		Cyclophilin qR	CAAAAACTCCTCTGCCCCAATCAA
Tubulin	AT1G50010	Tubulin qF	ATGTGGGTCAGGGTATGGAA
		Tubulin qR	CCGACAACCTTCTTAGTCTCCTCT
GAPDH	AT3G26650	GAPDH qF	TGGTTGATCTCGTTGTGCAGGTCTC
		GAPDH qR	GTCAGCCAAGTCAACAACTCTCTG
FLA16	AT2G35860	FLA16 qF1	GGTCGGATTTCAGTTCAGGGT
		FLA16 qR1	CCCCATCATTCTACAAGCTACCT
		FLA16 qF2	TCCCACATAAAGTGTTGGCTCAAG
		FLA16 qR2	CTCTTCTTGATTTAGAAACTTTCTTAACG
***pFLA16*: FLA16 fusion proteins**			
pFLA16:FLA16	AT2G35860	pFLA16_F	TTCGGTACCAGTCAACATTC
		SP16_F	CCAGTACCGGGTCAAATAA
		SP16_R	TTATTTGACCCGGTACTGG
		FLA16_R	CCATGGAGTGGATCCTCA
pFLA16:FLA16-VH			
		VENUS_F	GTGAGCAAGGGCGAGGAG
		VENUS_R	CTTGTACAGCTCGTCCATGCC
		16RVH_F	TAGCTTGTAGAATGATGGGGTCAC
		16RVH_R	ATATCTCATTAAAGCAGGACTCTAGA
		FLA16_His R	CTCTAGAGGATCCTCAATGATGATGGT
**Genotyping**			
fla16	AT2G35860	LBb1.3	ATTTTGCCGATTTCGGAAC
		PEL163ii_F	AAGCTTTTCATCACAAATAAACCATGC
		PEL164_R	AATAGGCACACAAATGGATCC

2. Supplementary Figures

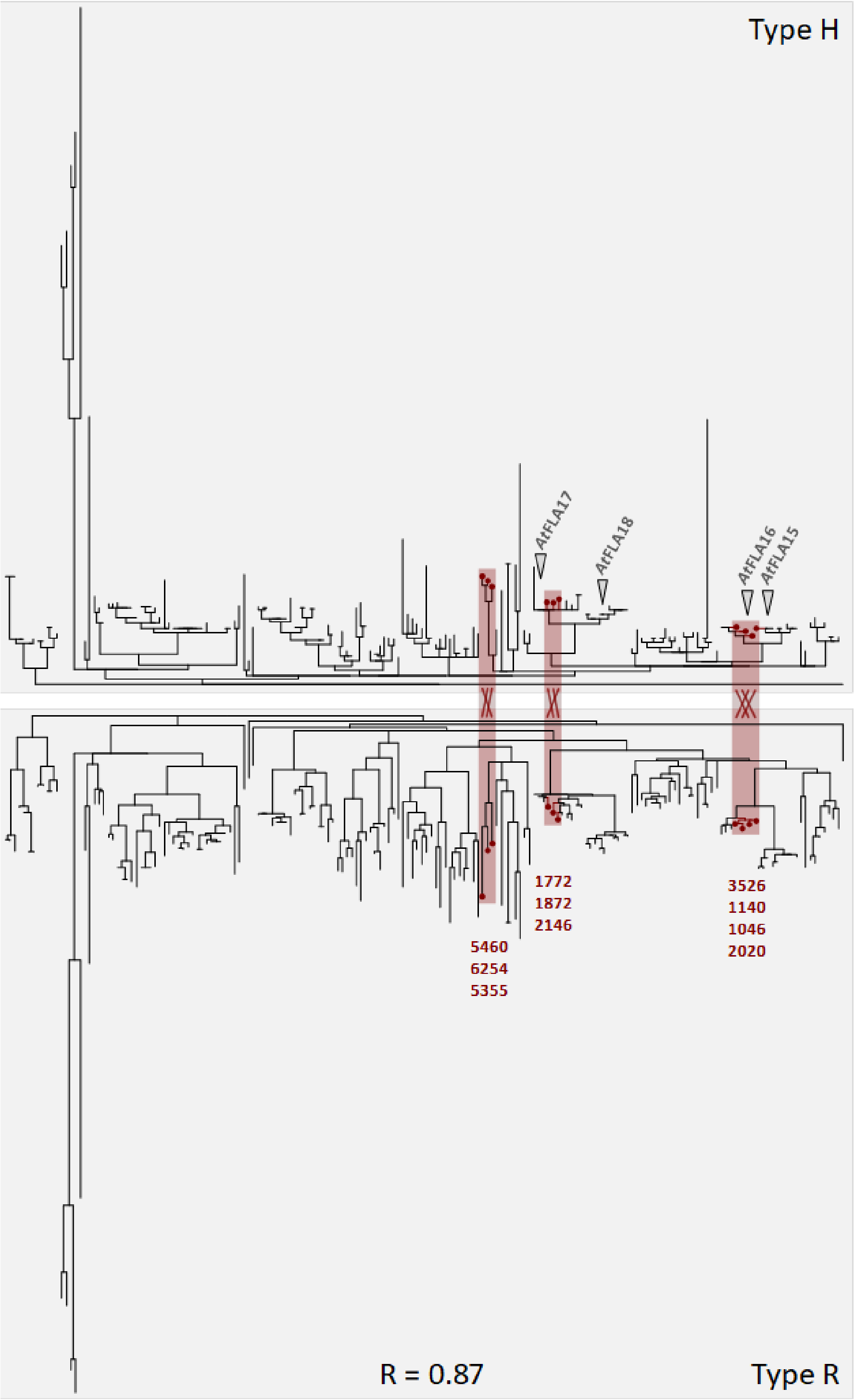

Supp Figure S1. Phylogeny of fasciclin domain types R-H in Group B FLAs. Maximum likelihood phylogeny of FLA sequences that contain fasciclin domain type R-H (Shafee et al. 2020). A clade of algal members has been omitted due to very long branch lengths. Nodes with <50% bootstrap support collapsed (demonstrating high overall bootstrap support). Red shading indicates branches where R and H domain phylogenies do not match and the corresponding sequence numbers. *At*FLA15 to 18 indicated by arrowheads. Sequences named as per **Supplementary data file 1**.

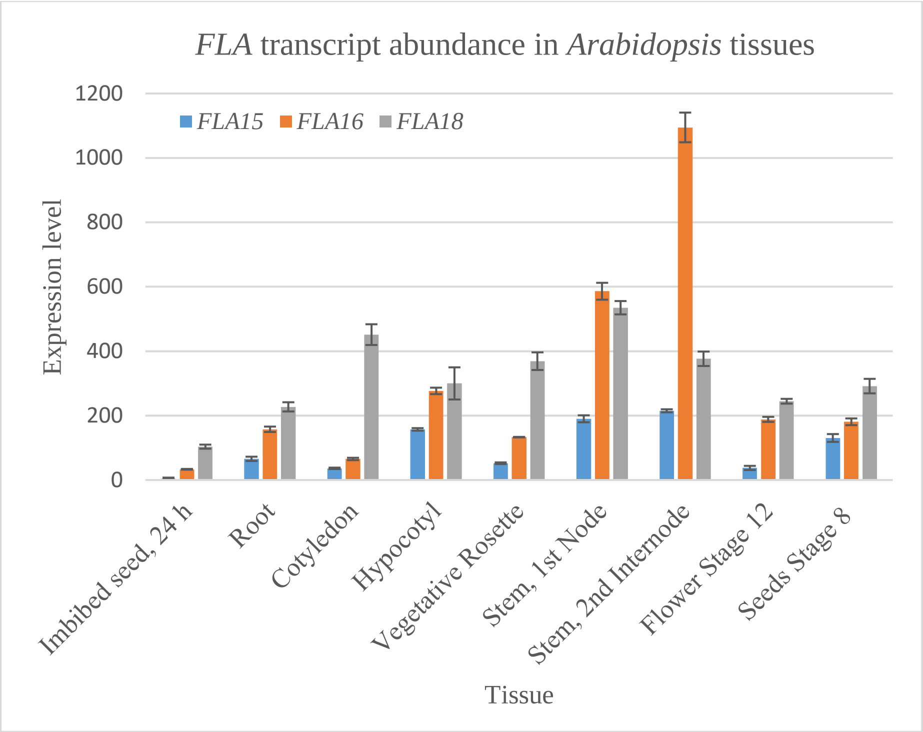

Supp Figure S2. Expression level of *FLA15*, *FLA16* and *FLA18* in selected *Arabidopsis* tissues. *Arabidopsis* developmental data set of (Schmid et al. 2005) with data extraction tool by (Toufighi et al. 2005; Winter et al. 2007).

A

>AT2G35860.1|FLA16

MDSSYGATKFLLLLFLTTSIATALPDNKPVPGQINSNSVLVALLDSHYTELAELVEKALLLQTLEEAVGKH**NIT**IFAPRNDALERNL
DPLFKSFLLEPRNLKSLQSLLMFHILPKRITSPQWPSLSHHHRTLSNDHLHLTVDVNTLKVDSAEIIRPDDVIRPDGIIHGIERLLIP
RSVQEDFNRRRSLRSISAVIPEGAPEVDPRTHRLKKPSPAVPAGAPPVLPIYDAMSPGPSLAPAPAPGPGGPRGHFNGDAQVK
DFIHTLLHYGGYNEMADILV**NLT**SLATEMGRLVSEGYVLTVLAPNDEAMAKLTTDQLSEPGAPEQIMYYHIIPEYQTEESMYNA
VRRFGKVKYDSLRFPHKVLAQEADGSVKFGHGDGSAYLFDPDIYTDGRISVQGIDGVLFPKEETPATEIKPAAPVVKKVSKSRR*
GKLMEVACRMMGSRFIPCQ

B

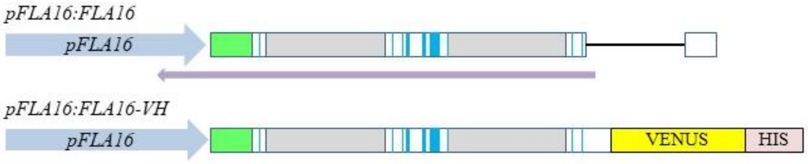

Supp Figure S3. Protein sequence and predicted domains of *Arabidopsis* FLA16 and schematic of FLA16 reporter constructs.

(A) The protein encoded by *Arabidopsis FLA16* is predicted to have a N-terminal signal peptide (green), two fasciclin1 (FAS1) domains (grey) with glycomotifs for *N*-linked glycans (underlined) and regions with AGP-like glycomotifs (blue) that are predicted to direct addition of large *O*-linked glycans. The position of an intron is indicated by *.

(B) Constructs used to complement the *fla16* mutant and as reporter constructs for *FLA16* expression and localization. The *pFLA16* promoter includes a 2347bp fragment upstream of the genomic DNA sequence of *FLA16.* Indicated underneath the *pFLA16*:*FLA16* construct by a purple arrow is an overlapping natural antisense transcript (At2G35859). FLA16 protein fusions included the *pFLA16* promoter, the *FLA16* coding region fused to the YFP variant VENUS, and HIS (*pFLA16:FLA16-VH*).

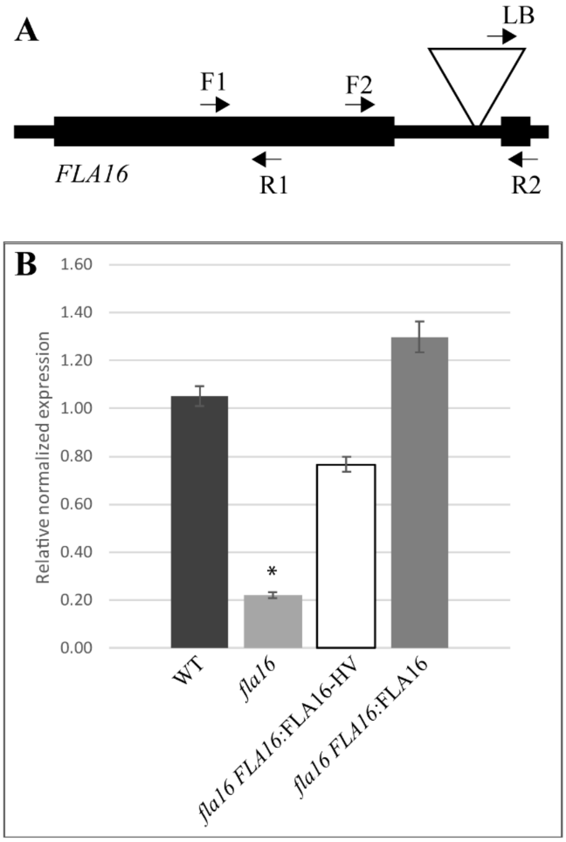

Supp Figure S4. Schematic of the T-DNA insertion in *FLA16* and transcript levels of *FLA16* in stems of *Arabidopsis*.

(**A**) The position of the T-DNA insertion (triangle) in the intron of *FLA16* and primers used for Q-PCR analysis (F1/R1) and genotyping (F2/R2) are indicated.

(**B**) *FLA16* transcript levels are significantly reduced in stems of a *fla16* mutant compared to WT. *FLA16* transcript levels in *fla16* mutants complemented with *pFLA16:FLA16* and *pFLA16:FLA16-VH* transgenes are 73% and 123% of WT levels, respectively. Transcript levels were determined relative to DNA standards of known concentration and normalized with GAPDH, tubulin and cyclophilin housekeeping genes (Czechowski et al. 2005). N = 3 biological replicates, for *pFLA16:FLA16* and *pFLA16:FLA16-VH* two independent transformed lines were used with 3 biological replicates for each. $p<0.05$ (*) using Student t test, data are represented as mean ± SE.

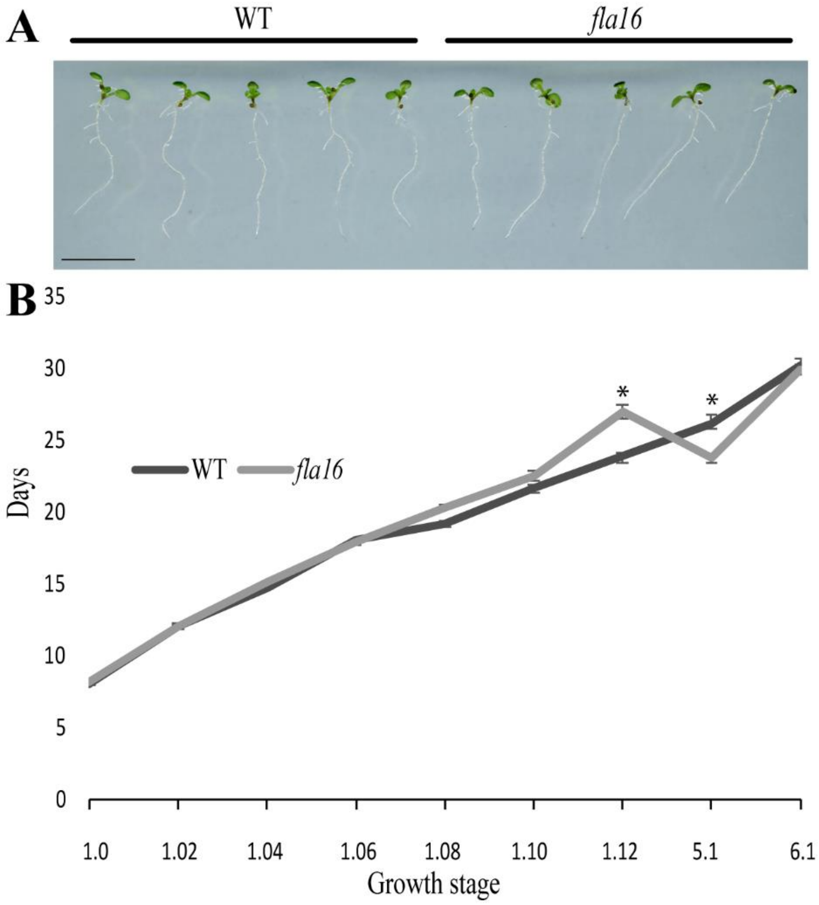

Supp Figure S5. Phenotyping of WT and *fla16* mutants at selected growth stages.
(A) WT and *fla16* mutant 7 day old seedlings (Stage 1.0 (Boyes et al. 2001a)) show no obvious differences in growth.
(**B**) Growth of WT and *fla16* mutants shows no significant difference until growth stage 1.12. The first flower bud visible (stage 5.1) occurs earlier in *fla16* which flower at the 12 rosette leaf stage (1.12) and WT at the 14 rosette leaf stage. No difference in the time of first flower emergence (Stage 6.1) was observed. (*) Statistically significant value at $p<0.05$ using a Student t test (see Supplementary Table S1). Error bars indicate ± SE. N = 48-50 biological replicates

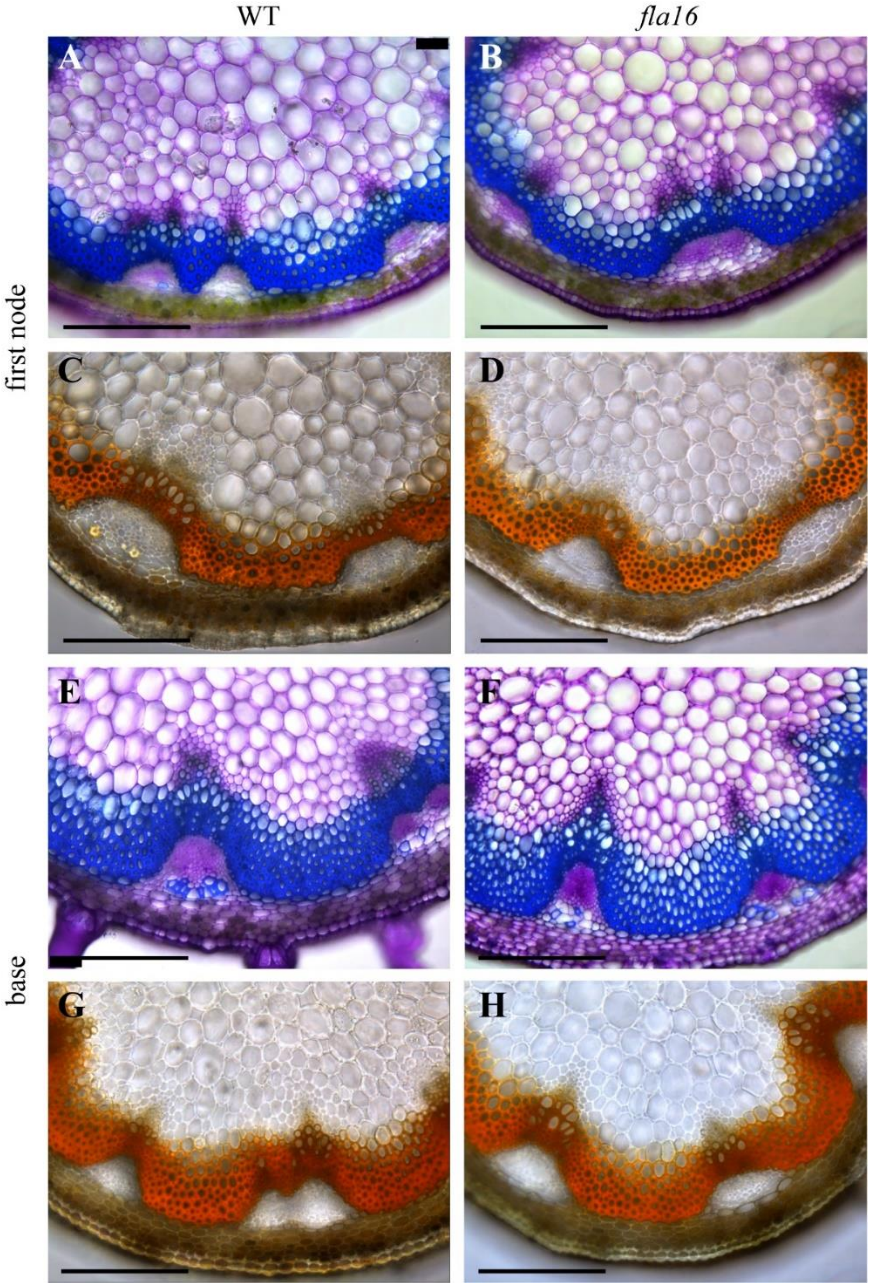

Supp Figure S6. Cellular morphology in transverse sections of WT and *fla16* mature stems stained with toluidine blue and Mäule reagent.

Hand sections of fresh stems at growth stage 6.5 (Boyes et al. 2001b) were taken at the first node (**A-D**) and base (**E-H**) of WT and *fla16* stems and stained with toluidine blue (**A, B, E, F**) or Mäule reagent (**C, D, G, H**). No obvious differences in either cellular morphology or lignin distribution are observed in the *fla16* mutant (**B, D, F, H**) compared to WT (**A**, **C, E, G**). Scale bar=200 µm.

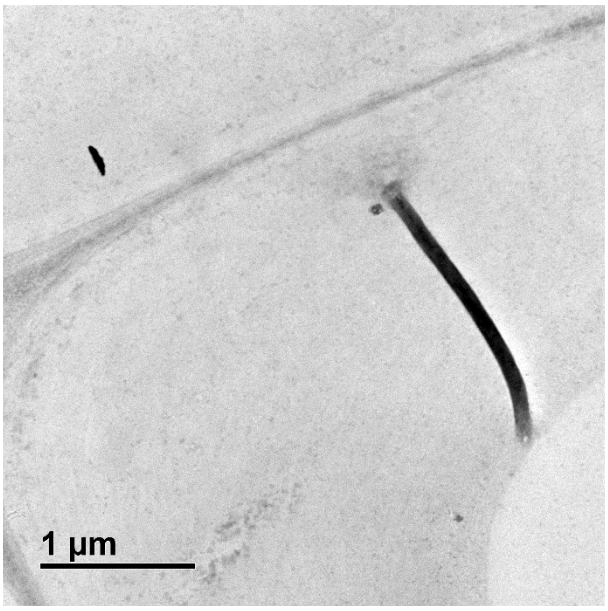

Supp Figure S7. Transmission electron micrograph image of interfascicular fibre cells at the stem first internode of *pFLA16*:FLA16-VH in WT. Gold labelling was not observed in controls (N = 5 cells, 2 biological replicates) lacking the anti-HIS antibody.

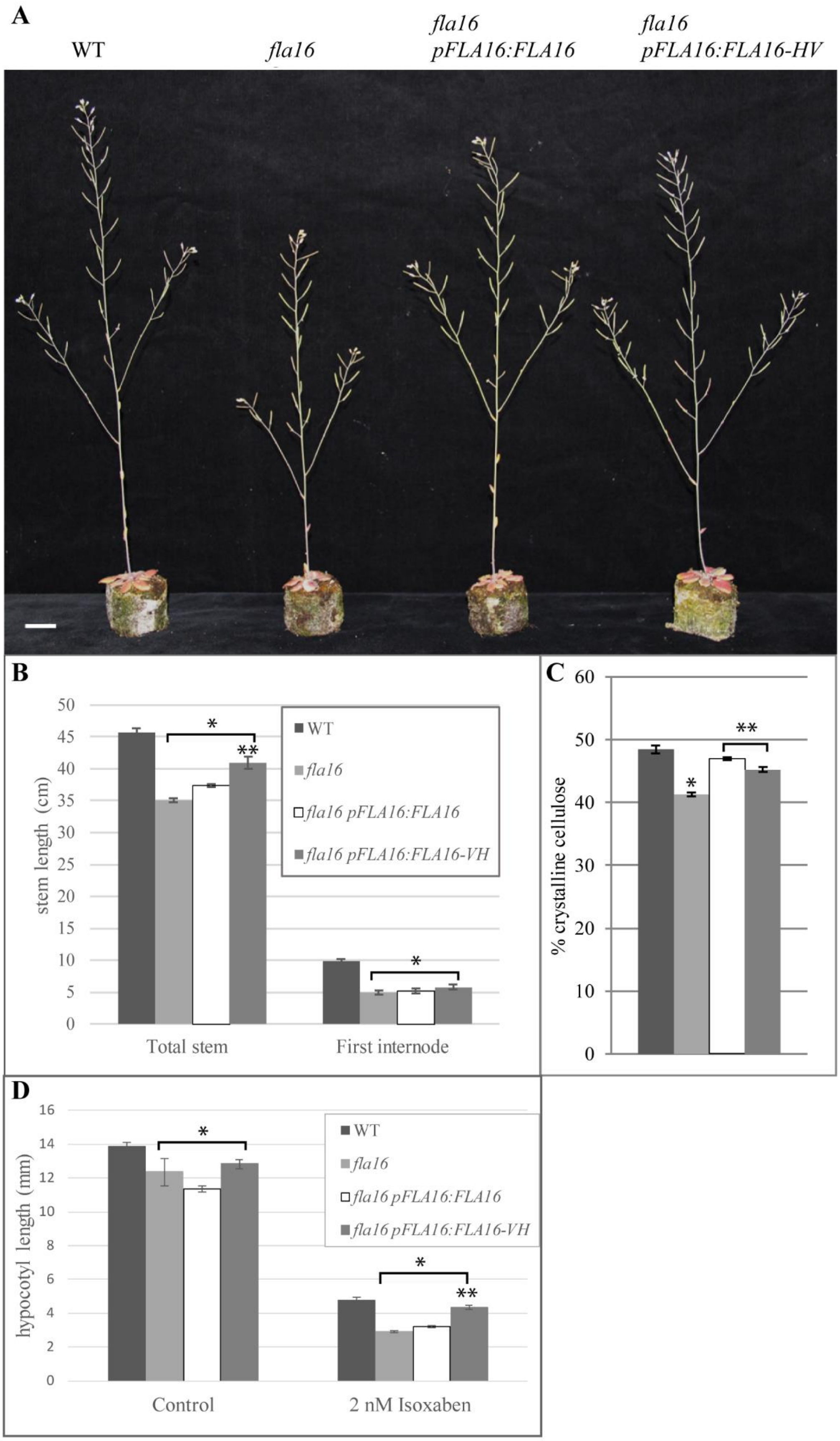
A
WT
fla16
fla16 pFLA16:FLA16
fla16 pFLA16:FLA16-HV
B
stem length (cm)
50
45
40
35
30
25
20
15
10
5
0
Total stem
First internode
*
**
WT
fla16
fla16 pFLA16:FLA16
fla16 pFLA16:FLA16-VH
C
% crystalline cellulose
60
50
40
30
20
10
0
*
**
D
hypocotyl length (mm)
16
14
12
10
8
6
4
2
0
Control
2 nM Isoxaben
*
**
WT
fla16
fla16 pFLA16:FLA16
fla16 pFLA16:FLA16-VH

Supp Figure S8. Complementation of the *fla16* mutant with *pFLA16:FLA16* and *pFLA16:FLA16-VH*.
Total stem length was partially recovered in both *fla16 pFLA16:FLA16* (81.7%) and *fla16 pFLA16:FLA16-VH* (89.5%) complementation lines with no recovery of first internode length. N = 25 (WT, *fla16*) or 40 (*fla16 pFLA16:*FLA16, *fla16 pFLA16:*FLA16-VH) biological repeats, $p<0.05$ using Student t test compared to WT (*) and *fla16* (**) (**A, B**). Data are presented as means ±SE. **C**. Crystalline cellulose levels are largely recovered to WT levels in both *fla16 pFLA16:FLA16* (98.2%) and *fla16 pFLA16:FLA16-VH* (93.6%) complementation lines. N = 2 (WT, *fla16*) or 6 (*fla16 pFLA16:*FLA16, *fla16 pFLA16:*FLA16-VH) biological repeats with two technical repeats each. Scale bar = 1cm **D.** Significantly reduced hypocotyl length is observed in *fla16 pFLA16:FLA16* (82%) and *fla16 pFLA16:FLA16-VH* (93%) on MS media plates (control) compared to WT (*) with no recovery of the *fla16* mutant. Partial recovery of hypocotyl length is observed in *fla16 pFLA16:FLA16-VH* treated with 2 nM isoxaben compared to the *fla16* mutant (**) (**A**, **B**). N = 40-60, data represented as mean ± SE, $P < 0.05$ using Student t test.

References

Boyes DC, Zayed AM, Ascenzi R, McCaskill AJ, Hoffman NE, Davis KR and Gorlach J (2001a) Growth stage-based phenotypic analysis of Arabidopsis: a model for high throughput functional genomics in plants. **Plant Cell** 13: 1499-1510
Boyes DC, Zayed AM, Ascenzi R, McCaskill AJ, Hoffman NE, Davis KR and Görlach J (2001b) Growth stage–based phenotypic analysis of Arabidopsis: a Model for high Throughput functional genomics in plants. **Plant Cell** 13: 1499–1510
Czechowski T, Stitt M, Altmann T, Udvardi MK and Scheible W-R (2005) Genome-Wide Identification and Testing of Superior Reference Genes for Transcript Normalization in Arabidopsis. **Plant Physiology** 139: 5-17
Schmid M, Davison TS, Henz SR, Pape UJ, Demar M, Vingron M, Scholkopf B, Weigel D and Lohmann JU (2005) A gene expression map of Arabidopsis thaliana development. **Nat Genet** 37: 501-506
Shafee T, Bacic A and Johnson K (2020) Evolution of Sequence-Diverse Disordered Regions in a Protein Family: Order within the Chaos. **Mol Biol Evol** 37: 2155-2172
Toufighi K, Brady SM, Austin R, Ly E and Provart NJ (2005) The Botany Array Resource: e-Northerns, Expression Angling, and promoter analyses. **Plant J** 43: 153-163
Updegraff DM (1969) Semimicro determination of cellulose in biological materials. **Anal Biochem** 32: 420-424
Winter D, Vinegar B, Nahal H, Ammar R, Wilson GV and Provart NJ (2007) An "Electronic Fluorescent Pictograph" browser for exploring and analyzing large-scale biological data sets. **PLoS One** 2: e718
